# Receptors and Host Factors for Enterovirus Infection: Implications for Cancer Therapy

**DOI:** 10.3390/cancers16183139

**Published:** 2024-09-12

**Authors:** Olga N. Alekseeva, Le T. Hoa, Pavel O. Vorobyev, Dmitriy V. Kochetkov, Yana D. Gumennaya, Elizaveta R. Naberezhnaya, Denis O. Chuvashov, Alexander V. Ivanov, Peter M. Chumakov, Anastasia V. Lipatova

**Affiliations:** 1 Engelhardt Institute of Molecular Biology, Russian Academy of Sciences, 119991 Moscow, Russia; olga_aleks@eimb.ru (O.N.A.); pavel.gealbhain@gmail.com (P.O.V.); dvkochetkov@gmail.com (D.V.K.); yanagumenny@mail.ru (Y.D.G.); elizaveta2001@gmail.com (E.R.N.); nidhegg04@gmail.com (D.O.C.); chumakovpm@yahoo.com (P.M.C.); 2 Department of Molecular Microbiology and Immunology, Norris Comprehensive Cancer Center, Keck School of Medicine, University of Southern California, Los Angeles, CA 90033, USA

**Keywords:** enterovirus, CRISPR/Cas9 technology, oncolytic virus, echovirus, coxsackievirus, viral receptors

## Abstract

**Simple Summary:**

Enteroviruses are considered to be promising oncolytic agents towards a variety of human cancers. Enteroviruses comprise polioviruses, coxsackieviruses, and echoviruses. Their efficacy depends on their ability to infect respective tumor cells. As non-enveloped viruses, they can enter cells via different receptors, many of which are expressed in tumors at higher levels. However, understanding the precise role of individual receptors in virus entry is complicated, and often requires the development of knockdown/knockout models. In this review we summarize current concepts about the roles of individual receptors in the entry of enteroviruses into cells, as well as the impact of host RNA-sensing machinery that can activate interferon signaling in response to viral infections. Several other host cell factors are also discussed.

**Abstract:**

Enteroviruses, with their diverse clinical manifestations ranging from mild or asymptomatic infections to severe diseases such as poliomyelitis and viral myocarditis, present a public health threat. However, they can also be used as oncolytic agents. This review shows the intricate relationship between enteroviruses and host cell factors. Enteroviruses utilize specific receptors and coreceptors for cell entry that are critical for infection and subsequent viral replication. These receptors, many of which are glycoproteins, facilitate virus binding, capsid destabilization, and internalization into cells, and their expression defines virus tropism towards various types of cells. Since enteroviruses can exploit different receptors, they have high oncolytic potential for personalized cancer therapy, as exemplified by the antitumor activity of certain enterovirus strains including the bioselected non-pathogenic Echovirus type 7/Rigvir, approved for melanoma treatment. Dissecting the roles of individual receptors in the entry of enteroviruses can provide valuable insights into their potential in cancer therapy. This review discusses the application of gene-targeting techniques such as CRISPR/Cas9 technology to investigate the impact of the loss of a particular receptor on the attachment of the virus and its subsequent internalization. It also summarizes the data on their expression in various types of cancer. By understanding how enteroviruses interact with specific cellular receptors, researchers can develop more effective regimens of treatment, offering hope for more targeted and efficient therapeutic strategies.

## 1. Introduction

Enteroviruses are small RNA viruses with non-enveloped virions and a single-strand positive RNA genome comprising approximately 7400 nucleotides [1]. They have attracted much attention due to a wide array of clinical manifestations in humans, ranging from mild symptoms to severe diseases like poliomyelitis and viral myocarditis [2]. Several enteroviruses also belong to a group of zoonotic infections [3]. Their simple, structured genomes are the perfect models for the investigation of fundamental virology and molecular biology [4]. Enteroviruses exhibit remarkable genetic flexibility through frequent mutations and recombination, allowing further investigation of viral evolution and adaptation [5].

Enteroviruses are classified into fifteen species based on their structural similarity (Enteroviruses A to L, rhinoviruses A, B, and C); however, many of them retain their original names, like Echovirus 1 and Coxsackievirus A7 [1,6,7,8,9,10,11]. Most enterovirus strains are non-pathogenic. This could be exemplified by the isolation of these viruses from the stool of healthy children or even the usage of certain strains as live enterovirus vaccines (LEVs) [12] for non-specific prevention of viral infections [13,14]. Importantly, enteroviruses can also exhibit oncolytic activity [15]. The most prominent example is the bioselected strain of a non-pathogenic Echovirus type 7/Rigvir that was approved for melanoma treatment, although currently its license is suspended [16,17]. Several other enteroviruses, including the non-pathogenic Coxsackievirus A21 strain CAVATAK and the recombinant poliovirus PVSRIPO, are also currently being evaluated as anticancer agents [18,19,20].

**Figure 1:**
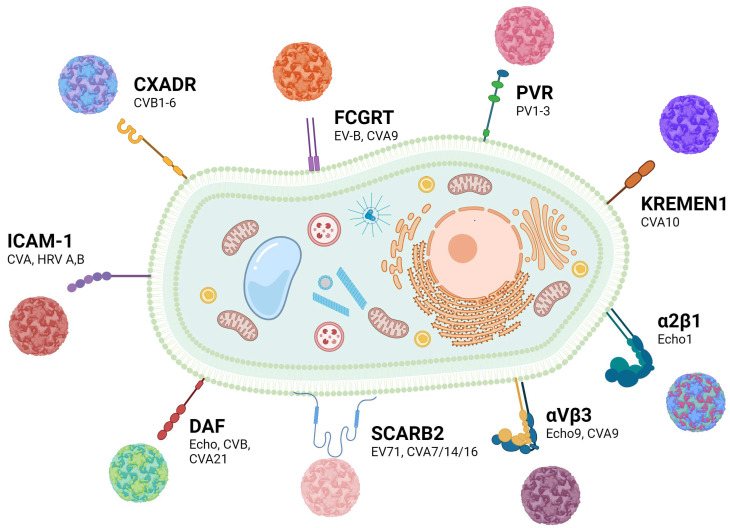
Receptors used by enteroviruses for cell entry.

The sensitivity of cells to enteroviruses partly depends on the ability of the agents to exploit specific receptors and coreceptors for viral entry (Table 1) [21,22]. These receptors facilitate virus binding and capsid destabilization, while coreceptors assist in virus binding and interaction with the receptor or any other uncoating factors [23]. Most receptors are glycoproteins, and their carbohydrate components affect interaction with virus particles [7,24]. Many enteroviruses can bind to several alternative cellular receptors for internalization and subsequent replication (Figure 1), thus ensuring the infection of various types of cells. For this reason, enteroviruses have attracted special attention as agents for personalized cancer therapy, as the tumors that do not respond to treatment with one oncolytic enterovirus could still be sensitive to another enterovirus strain that enters cells via an alternative mechanism. So, the development of anticancer agents based on enteroviruses requires an understanding of the mechanisms of their entry into tumor cells, and thus, of the usage of specific cell receptors.

One of the major directions in the development of more effective oncolytic viruses is the enhancement of their oncoselectivity by introducing modifications into their genomes. These modifications include the deletion of viral genes that confer pathogenicity of these infectious agents, alteration of structural proteins to potentiate binding of virions to tumor cells, and expression of factors that enhance the clearance of tumor cells by the host immune system [25,26,27,28,29]. Such approaches are generally applicable to viruses with large genomes including herpesviruses, poxviruses, and rhabdoviruses (particularly to vesicular stomatitis virus) [30,31,32]. However, in the case of enteroviruses, whose genome encodes a peptide of just 2000 amino acids, this approach is much less efficient, as deletions/insertions profoundly reduce virus fitness. Nevertheless, there are a few successful examples. The first one is Rigvir that was obtained by passaging Echovirus 7 on melanoma cells with a suppressed metabolism [33]. Such passaging resulted in several mutations in core proteins that affected the efficiency of virus entry and replication. Another example is the bioselected coxsackievirus B6 obtained by our group that can effectively replicate in various types of tumor cells [34]. Finally, we recently reported the adaptation of Echovirus 1 to glioblastoma and melanoma cells [35]. However, the problem of attenuation of virus internalization remains mostly unexplored.

This review aims to present the current knowledge on the interaction of various enteroviruses with host cell receptors. As non-enveloped viruses may use different receptors, we specifically analyzed literature data on the attachment and internalization of enteroviruses into cells with downregulated expression of specific receptors using CRISPR/Cas9 technology and other approaches. Furthermore, we discuss the existing data on the expression of these receptors in various tumors. So, this review should broaden our understanding of the mechanisms of enterovirus infection and contribute to the development of more efficient oncolytic enteroviruses.

**Table 1:** Cell receptors used by enteroviruses and mechanisms of their internalization.

Receptor	Virus	Mechanism of Entry	References
PVR	Poliovirus types 1, 2, 3	Receptor-mediated endocytosis	[36,37,38,39,40,41]
FCGRT	Echovirus 1, 3, 6, 7, 9, 11, 13, 14, 18, 25, 26, 30, Coxsackievirus A9, EV-B85	Caveolar-mediated endocytosis/receptor-mediated endocytosis	[42,43,44]
SCARB2	EV71, Coxsackievirus A7, A10 A14, A16	Clathrin-mediated endocytosis	[45,46,47,48]
Integrins	Echovirus 1, 5, 8, 9, 22, Coxsackievirus A9, B1	Caveolar-mediated endocytosis	[49,50,51,52,53]
KREMEN1	Coxsackievirus A10,	Caveolin-dependent mechanism	[54,55]
ICAM-1	Coxsackievirus A21	Receptor-mediated endocytosis	[56,57]
CAR	Coxsackievirus B1, B2, B3, B4, B5, B6	Receptor-mediated endocytosis	[58,59,60,61,62]
DAF	Echoviruses 6, 7, 11, 12, 20, 21, 70, and D68 (Probable), Coxsackievirus A21, B1, B3, B5	Receptor-mediated endocytosis	[63,64,65,66,67,68,69,70,71,72]

## 2. Poliovirus Receptor

The poliovirus receptor (PVR/CD155), a transmembrane immunoglobulin-like glycoprotein, plays a crucial role in cell-to-cell interaction. PVR interacts with other members of the nectin family as well as with IgSF molecules (TIGIT, CD226, CD96), modulating the immune response [73,74,75]. This interaction activates intracellular signaling, leading to IL-10 production by dendritic cells [76,77].

PVR comprises three extracellular Ig-like domains: a V-type domain and two C2 domains [78]. The N-terminal Ig-like domain (D1) is essential for the attachment of poliovirus particles [79,80,81].

PVR binds to poliovirus, interacting with the capsid’s “canyon” region [82,83]. It leads to changes in virion structure and the consequent release of myristoylated VP4 molecules and RNA. This process involves a “pocket factor” lipid molecule in the canyon, facilitating further capsid transformations and direct binding to the cell membrane [6]. The PVR binds a virion with an oblique angle, allowing its D1 domain to enter the canyon and connect with the capsid surface near the center of the icosahedral asymmetric unit [37]. The PVR-binding site is composed of residues of VP1, VP2, and VP3 capsid proteins. On the capsid surface, PVR leaves a distinctive imprint consisting of three separate patches, two of which resemble the attachment pattern of an intercellular adhesion molecule-1 on rhinovirus.

RNA penetration into the cytosol occurs at neutral pH, thus being independent of endosomal internalization [38]. However, internalization traits might vary depending on cell type. For example, in brain endothelial cells, poliovirus binding to PVR induces the rearrangement of the cytoskeleton required for caveolin-dependent entry of the virus [84].

PVR is absolutely crucial for poliovirus, as a pooled sgRNA CRISPR/Cas9-mediated gene knockout in HeLa cells was shown to have led to a complete resistance to poliovirus type 1 infection [85]. This was later confirmed in rhabdomyosarcoma cells (RD) [86]. At the same time, knockout of the PVR gene does not affect the sensitivity of cells to other enteroviruses, indicating that PVR is strictly specific for polioviruses [21]. This is supported by the well-known fact that poliovirus infection typically occurs only in human and primate cells, whereas it can also replicate in rat cells expressing human PVR [87]. So, in the case of resistance of tumor cells to poliovirus, other oncolytic enteroviruses could be used.

## 3. Intercellular Adhesion Receptor 1

Intercellular Adhesion Molecule 1 (ICAM-1), a member of the immunoglobulin superfamily, comprises 505 amino acids [62,74]. Due to tissue-specific glycosylation, this protein has an apparent molecular weight ranging from 76 to 114 kDa. ICAM-1 consists of five extracellular Ig-like domains, a transmembrane region, and a 28 amino acid cytoplasmic domain [62]. ICAM-1, present on the surface of endothelial and epithelial cells, fibroblasts, and specific hematopoietic cells [75,76,77], acts as a ligand for leukocyte integrins and a receptor for fibrinogen [74,78]. ICAM-1 facilitates the entry of Coxsackievirus A21, Enterovirus 71, and other enteroviruses [21,87], including rhinoviruses [88]. In addition, ICAM-1 is exploited by rhinoviruses [88]. Virions bind ICAM-1 by the “canyon” groove of their capsid, causing the release of the “pocket factor” and subsequent release of viral RNA [88]. After attachment to ICAM-1, enteroviruses are internalized via endocytosis. A noteworthy point is that ICAM-1 is also engaged in cell-to-cell virus spread [89].

The formation of the CVA21/ICAM-1 complex involves the interaction of the ICAM-1 D1 domain with specific regions of the VP1, VP2, and VP3 viral proteins [57]. The D-E loop, B-C loop, and G-F loop of the ICAM-1 D1 domain form contacts with the VP1 βG and βH strands, as well as with the VP1 G-H loop. In addition, the βG strand of the ICAM-1 D1 domain interacts with the VP1 E-F loop and the VP3 G-H loop. This is further enhanced by the connection of the βD and βE strands of the same ICAM-1 domain with the puff region of VP2. Poliovirus has a different structure of the VP2 puff region and the VP1 G-H loop, making interaction with ICAM-1 impossible.

## 4. Scavenger Receptor B2

Scavenger Receptor Class B Member 2 (SCARB2), also known as the Lysosomal Integral Membrane Protein II (LIMP-2) or CD36-like protein-2, is another receptor for enteroviruses. It is recognized by the coxsackievirus species A7, A14, and A16, as well as by EV71 [48,90,91]. This member of the CD36-like scavenger receptor family is a glycosylated type III transmembrane protein comprising 478 amino acids [91,92,93]. SCARB2 consists of an N-terminal transmembrane domain, an extracellular domain, a C-terminal transmembrane domain, and a cytoplasmic tail [94].

Mutations leading to SCARB2 inactivation in mammals confer heavy syndromes, including peripheral neuropathy and renal failure syndrome, characterized by myoclonus [95]. Deletion of its gene is related to Gaucher disease [96]. SCARB2 controls cell metabolism, as its deletion results in reduced lipid storage and a disbalance between glycolysis and oxidative phosphorylation [97]. In addition, it plays a role in endosome biogenesis and cholesterol trafficking [94]. SCARB2 expression in neural tissues explains EV71 neurotoxicity [48,91,98]. The protein has ten N-glycosylation sites, though glycosylation is not essential for interacting with EV71 [48,99]. Amino acid residues 142–204 of SCARB2 are critical for EV71 attachment and cellular infection [48,99]. Models of the EV71-SCARB2 complex demonstrate that the hydrophobic pocket of the EV71 VP1 capsid protein is positioned next to the lipid-transfer tunnel of SCARB2 [90]. In this proposed binding arrangement, helices α_4_ and α_5_ from SCARB2′s viral binding domain are inserted into the EV71 capsid canyon, flanked by helices α_7_ and α_15_ in proximity of the virus five-fold and three-fold axes, respectively. This alignment correctly positions the conserved residues such as N102, D219, and K285 of VP1, and R182 and D183 of VP3 for interaction with domain III of the SCARB2 protein. Upon virus binding to SCARB2, EV71 undergoes structural changes facilitated by interaction with other receptors like PVR, CXADR, or ICAM-1. Virus RNA is released via the “lipid channel” in acidified endosomes, leading to VP4 release and A-particle formation, which is essential for virus replication [90,100].

SCARB2 is one of the key receptors for EV71, as both attachment and replication of this virus are significantly reduced after SCARB2 knockout [100,101,102]. SCARB2 importance is further exemplified by the increased susceptibility to EV71 of initially non-permissive mouse cells with ectopic SCARB2 expression [103]. CRISPR/Cas9-generated knock-in mice expressing human SCARB2 gain typical severe symptoms of EV71 infection [46,104,105]. A genome-wide CRISPR/Cas9 screen confirmed that SCARB2 is essential for EV71 infection but also identified SLC35B2 and B3GAT3 as additional host factors [106]. This work revealed that SCARB2 activity as a virus receptor depends on its sulfatation, with SLC35B2 regulating this process. Additionally, SCARB2 plays an important role in Coxsackievirus A10 (CVA10) infection, as siRNA-mediated suppression of SCARB2 expression in RD cells was shown to lead to a marked decrease in CVA10 VP1 expression after infection [47].

## 5. Integrins

Integrins are transmembrane proteins essential for cell adhesion and binding to various ligands, including extracellular matrix proteins and various plasma proteins [107]. Integrins are composed of α and β subunits, with vertebrates expressing eighteen α-subunits and eight β-subunits [107,108,109]. The subunits, consisting of about 1000 and 750 amino acid residues, are composed of multiple domains: an α-helical transmembrane region and an unstructured cytoplasmic tail.

The activity of α-subunits depends on chelating magnesium and calcium ions at specific sites. At the same time, the extracellular region of β-subunits has a cysteine-rich domain and binds bivalent cations [110]. The N-terminal region of integrin interacts with matrix glycoproteins that display an RGD motif, typical for such ligands as fibrinogen and fibronectin. The cytoplasmic domain is connected to the cytoskeleton via talin and vinculin proteins [107,108,109]. Expressed on numerous types of cells, integrins facilitate gene expression, signaling, cell proliferation, and differentiation [107,110].

Enteroviruses, such as Echovirus 1, 9, and Coxsackievirus A9, utilize integrins as receptors. The α_2_ß_1_ integrin (Very Late Antigen 2, VLA-2) binds Echovirus 1 through an RGD-independent mechanism [111], while a_v_ß_3_ and a_v_ß_6_ integrins serve as receptors for both Coxsackievirus A9 and Echovirus 9, with Coxsackievirus A9 requiring the RGD motif for interaction [111,112,113]. Other enteroviruses also utilize these integrins, despite an absence of the RGD motif [79]. The α_2_I domain binds within the canyon on the EV1 surface, forming extensive contacts with the outer canyon wall [80]. In such a docked position, both the N- and C-termini are exposed to solvent rather than to the virus surface. The α_3_ helix of the α_2_I domain and its connecting loops interact with the VP2 capsid protein of one protomer, while the opposite terminus contacts VP3 of a neighboring protomer.

The interaction of integrins with enteroviruses leads to virion structural rearrangement, resulting in the formation of A-particles. For instance, Echovirus 1 interaction with VLA-2 triggers caveolin-dependent endocytosis, with subsequent virion uncoating occurring in caveosomes [114]. The internalization signal is likely to be the virion-induced clustering of integrins that activates a cascade of protein kinases [6,81]. However, internalization mechanisms might vary depending on cell type, underlying different sensitivities to various enterovirus strains.

## 6. Coxsackievirus and Adenovirus Receptor

The Coxsackievirus and Adenovirus Receptor (CAR) is a transmembrane protein that belongs to the CTX family of the immunoglobulin superfamily (IgSF). It plays a significant role in cell adhesion [6,115,116,117,118,119,120,121]. The complete cellular function of CAR is not entirely understood yet. However, this receptor is involved in cell adhesion and recognition, particularly in homophilic interactions between epithelial cells [121]. CAR is abundantly present in normal and tumor tissues of various organs such as the heart, brain, stomach, prostate gland, and liver [10,115,120]. During embryogenesis, CAR expression is high in the central and peripheral nervous systems [118,119,120], skeletal muscles [121], and different types of epithelia [116,117].

Both human and mouse CAR proteins have an approximate molecular weight of 46 kDa and feature two disulfide bonds [119]. Human CAR, encoded by the CXADR gene, comprises 365 amino acids. It is organized into two extracellular immunoglobulin-like domains, a transmembrane domain, and an intracellular tail [115]. The D1 domain of CAR, which is capable of dimerization, is crucial for virus binding and facilitating intercellular adhesion [122]. Transcription and alternative splicing of the CXADR gene results in the expression of the two mRNA isoforms that encode structurally similar proteins that differ in the C-terminal PDZ segments [115,116,123].

CAR is the primary receptor for Coxsackie B viruses (CV-B1 to B6) and adenoviruses (types 2 and 5) [117,122,124]. Cardiac-specific CAR-knockout mice showed a significant reduction in the replication rate of Coxsackievirus B3 as well as of heart tissue damage caused by the infection. Moreover, knockout of the CXADR gene has demonstrated a loss of sensitivity to Coxsackievirus B5, confirming the vital role of this protein in cellular entry [125].

CAR interacts with viruses in the virion canyon area, triggering A-particle formation [126]. However, the uncoating process varies depending on the viral strain and cell type. For instance, Coxsackievirus B3 forms A-particles in acidified, clathrin-coated endosomes [127], whereas Coxsackievirus B4 is internalized in a clathrin-independent manner [126].

## 7. Decay Accelerating Factor

The Decay Acceleration Factor (DAF/CD55) is a member of a complement regulatory protein superfamily [128,129,130,131]. This 70 kDa protein comprises several short consensus repeat (SCR) domains [130,131]. DAF is anchored to the cell membrane via interaction with phosphatidyl-inositol groups. It is found on erythrocytes, platelets, and lymphocytes, as well as on various tumor and transformed cell lines [132]. DAF protects cells from complement-mediated lysis [128]. Besides, it is implicated in tumor progression, as it is highly expressed in cancer stem cells (i.e., tumor-initiating cells) and confers resistance to anticancer therapy [133].

DAF is recognized by numerous enteroviruses, including strains of Coxsackieviruses A21, B1, B3, and B5, or various echoviruses [67,68,69,71,134,135]. In particular, it is the main receptor for Rigvir [33]. The mechanism of virion binding to DAF is quite distinctive from other receptors, as it interacts not with the canyon region of a virus particle but stretches around the virion’s surface by forming an arch [6]. For this, DAF uses various SCR combinations and surface areas for Echoviruses 7, 12, and Coxsackievirus B3 [63]. The DAF/CD55 receptor interacts with Echovirus 6 mainly through its SCR3 and SCR4 domains [23]. In SCR4, an arginine residue at position 214 (R214) forms bonds with the T154 residue of the viral VP2 core protein, while aspartate D240 interacts with VP2′s threonine T163. The SCR3 domain interacts with both VP1 and VP2 core proteins through multiple amino acid residues.

The interaction of virions with the DAF receptor induces DAF clustering, activation of c-Abl tyrosine kinase, and subsequent cytoskeleton reorganization. This facilitates the movement of the virus–DAF complex towards CAR. Additionally, the Fyn protein kinase, a member of the Src family, enhances the access of a virus to CAR, subsequent formation of A-particles, and virion uncoating by the phosphorylation of caveolin [136]. It is also tempting to speculate that these clusters are formed on lipid rafts, as these rafts were shown to be involved in DAF-mediated internalization of EV11 [137]. However, it is worth noting that the specific internalization process depends on the viral strain and the cell type.

However, presence of DAF alone in many cases is not sufficient for viral infection, as it does not trigger virion uncoating. Instead, it acts rather as a co-receptor with other receptors on cell surface. For example, the entry of coxsackieviruses A also requires ICAM-1 [69,71] and possibly other viral receptors such as KREMEN 1. For coxsackieviruses B, CAR is essential [138]. According to current concepts, DAF ensures that viral particles concentrate on the cell surface to increase the probability of their interaction with the primary uncoating receptor. While CAR is located in tight junctions and less accessible in the intestinal epithelium [139], apical DAF protein could be more exposed on basal and lateral membranes of tumor cells [140]. This could resemble the case of receptors for hepatitis C virus: CD81 and SR-B1, acting as receptors on the basolateral membrane, are needed to transfer bound virions to claudin 1 and occludin receptors at the tight junctions [141,142]. It should be also discussed that DAF may act as a primary receptor for some strains of enteroviruses. This is supported by several findings for coxsackieviruses. First, overexpression of both DAF and ICAM1 diminishes virus replication levels, compared to overexpression of any single receptor [143]. Second, the bioselection of Coxsackievirus A21 in DAF-expressing but ICAM-1-negative cells by Johansson et al. yielded a strain that rapidly infects ICAM-1-deficient tumor cells [144]. However, it is worth noting that neither of these two studies evaluated the possibility of other co-receptors.

Nevertheless, it remains clear that DAF is one of the key factors for the lytic infection of coxsackieviruses [143,144]. This allows us to propose that the efficacy of oncolysis by coxsackieviruses may be modulated by drugs that decrease DAF expression, and non-steroid anti-inflammatory drugs (NSAID) in particular [145].

## 8. Kringle Containing Transmembrane Protein 1

Another receptor of coxsackieviruses is the KREMEN protein 1 (KRM1) [54]. Together with the KRM2 paralog, it is a high-affinity receptor for the Dickkopf proteins (DKKs), which are known inhibitors of the canonical Wnt signaling pathway, crucial for embryonic development [146,147,148]. KRM1 and KRM2 are type I transmembrane proteins with an ectodomain consisting of KR, WSC, and CUB domains, essential for inhibiting Wnt signaling [149,150].

KRM1 is specifically utilized as an entry receptor by CVA10, distinguishing it from other enteroviruses like EV71 and CVA16 [54]. KRM1 can bind directly to the virus canyon, triggering the release of the pocket factor and consequent changes in the viral capsid with a release of the viral genome [54,84]. This interaction also initiates viral uncoating by altering the structure of viral particles [55,151].

KRM1 binds across two adjacent asymmetric units (ASUs) via its KR and WSC domains, positioned above the canyon [151]. In the first ASU (VP1.1), interaction with KRM1 occurs through the EF loop (K161 and T163) and the GH gating loop (T209, S217, and T219) of VP1, creating binding patches on both sides of the canyon. The C-terminus of VP1.2 enhances the stability of the interaction by contacting the WSC domain of the receptor. Additionally, the KR domain interacts with the VP2.1 EF loop protrusion (138 to 143) and facilitates the formation of strong ionic bonds. The side chains of W94 and W106 in the KR domain form π-cation interactions with K140 of VP2.1. N140 and Y165 establish additional hydrogen bond and hydrophobic interactions with VP3.2.

Insights into the host genetic factors influencing virus resistance were gained through insertional mutagenesis, creating a large mutagenized HAP1 cell pool. KRM1 was identified in cells resistant to CVA10 as a protein playing an essential role in the infection. It was confirmed by generating KRM1-deficient HAP1 and HeLa cells using TALEN and CRISPR techniques [143]. This resistance was observed without significant response changes to siRNA-mediated KRM1 knockdown, indicating that the resistance phenotype is indeed due to the absence of KREMEN1 [47]. Interestingly, cell lines lacking KRM1 remain permissive to other enteroviruses like poliovirus type 1 and CVB1. Finally, we should mention that KRM1 is abundantly expressed in normal tissues, whereas its expression is much lower or even absent in many, if not most tumor cell lines [146]. This might limit the usage of enteroviruses that rely on KRM1.

## 9. Neonatal Fc Receptor

The Fc receptor and transporter for the IgG (FCGRT)–α-chain FcRn, also known as the Brambell receptor, is a versatile protein that, among its various functions, ensures the entry of Enterovirus B strains that do not use DAF/CD55 [23,43]. The FCGRT gene encodes FcRn that mediates the transfer of maternal gamma globulins to neonates, thus providing passive immunity during the early stages of immune system development [152]. Structurally, FcRn is a complex of an alpha chain encoded by the FCGRT gene (consisting of three extracellular domains, a transmembrane domain, and a short cytoplasmic tail) and the beta-2 microglobulin (B2M) [153,154]. FcRn also plays a role in protecting gamma globulin and albumin from degradation by binding to these proteins, thus prolonging their half-life in circulation and ensuring its functional efficacy [155,156,157]. Functionality of this receptor partially depends on glycosylation [158,159].

Elevated levels of FcRn have been reported for several types of tumors, including breast, ovarian, and lung cancers, where its expression has been associated with poor prognosis and disease progression [153,160,161,162,163]. FcRn can influence tumor evasion and metastasis [164]. It also modulates the activity of immune cells like NK cells and macrophages through IgG interactions, enhancing processes like antibody-dependent cell-mediated cytotoxicity (ADCC) and antibody-dependent cellular phagocytosis (ADCP), required for the elimination of tumor cells [165,166,167].

The role of FcRn as an enterovirus receptor was discovered in a study of Echovirus 6 (strain SJZ-366) via a CRISPR knockout screen in RD cells targeting genes of membrane proteins using an sgRNA library [99]. FcRn interacts with enteroviruses such as Echovirus 30 and enhances virus internalization after initial attachment to surface receptors like DAF or CAR [44]. However, other groups reported that DAF may not be involved in the FcRn-mediated entry of echoviruses [43]. According to current knowledge, FcRn is the key receptor for the entry of various types of echoviruses [168].

FcRn binds to Echovirus 6 virus via polar interactions with the α_2_ and α_3_ helices in the α_2_ domain, creating two interfaces on each side of the canyon [23]. Key residues like Q139, R140, Q143, and Q144 in the α_2_–α_3_ junction form hydrogen bond networks with the VP1, VP2, and VP3 subunits. Residues D145 and K146 interact in the canyon, forming bonds with VP1 subunit residues Y75, D91, and Q99, possibly crucial for transporting the “pocket factor”.

In summary, FcRn’s array of functions spans from its traditional role in immunity to its involvement in enterovirus infection and influence on immune cell functions, illustrating its importance in both viral pathogenesis and immune response mechanisms.

## 10. RIG-I and MDA5

**Figure 2:**
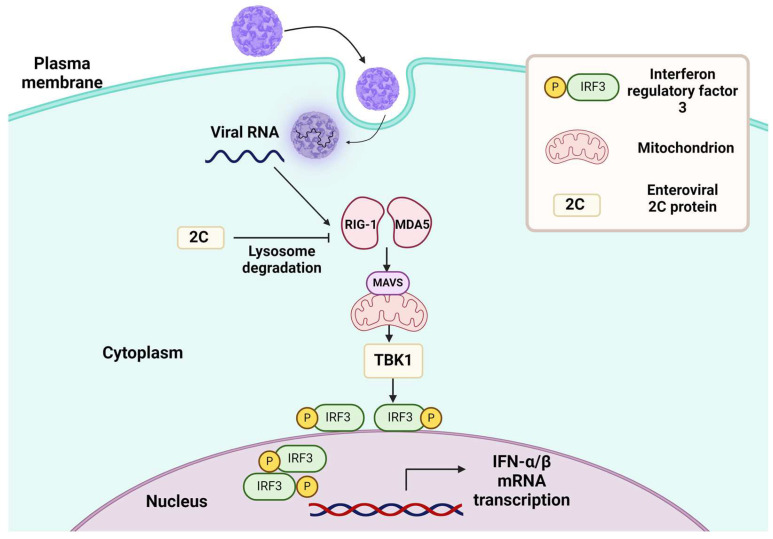
A schematic representation of interferon induction system activation during enteroviral infection.

The innate antiviral response activates RIG-I-like receptor (RLR) signaling, critically affecting virus entry [169]. Pattern recognition receptors detect molecular signatures associated with pathogens and initiate the production of type I and III interferons (IFN) to counteract viral invasion [170,171,172,173,174]. Two key sensors of viral RNA in human cells are the retinoic acid-inducible gene I (RIG-I) and melanoma differentiation-associated gene 5 (MDA5) [175]. Both of them contain two N-terminal caspase activation and recruitment domains (CARDs), a C-terminal regulatory/repressive domain, and a central DExD/H-box ATPase/helicase domain that detects viral RNA [176]. When enteroviruses infect the host cell, they release their RNA genome into the cytoplasm [177]. RIG-I identifies various features of viral RNA such as the triphosphate fragment on its 5′-terminus (5′-ppp) from de novo RNA synthesis and short double-stranded RNA (dsRNA) regions commonly found in single-stranded viral genomes [178]. When RIG-I binds to viral RNA, it undergoes conformational changes that expose its Caspase Activation and Recruitment Domains (CARD) motifs. This conformational shift enables RIG-I to interact with another crucial cellular component—the Mitochondrial Antiviral Signaling Protein (MAVS) (Figure 2) [179,180].

Different enteroviruses cleave MDA5, MAVS, and RIG-I in infected cells using their proteases: 2Apro digests MDA5 and MAVS, while 3Cpro targets RIG-I [181]. These cleavage events explain the downregulation of the IFN-α/β response in enterovirus-infected cells. In CVB3-infected cells, TBK1 phosphorylation and subsequent activation are suppressed, suggesting that this pathway is inhibited upstream of TBK1 [182]. The non-structural 2Cpro of CVA6 suppresses the production of IFN-β by targeting MDA5 and RIG-I to lysosomal proteases. This function is shared by EV71 and CVB3 but not by CVA16, suggesting that the latter may have an alternative way to stimulate virus replication. Also, 3Cpro of EV71 can inhibit IFN-β activation and RIG-I during viral infection. However, EV71 3Cpro does not exhibit inhibitory activity against MDA5 [183,184]. Structured RNA elements in the genome of CVB3 are recognized by MDA5 during infection and activate RIG-I if viral RNA retains 5′-ppp. The 5′ppp-containing cloverleaf (CL) RNA structure is a potent inducer of RIG-I, triggering a string antiviral response that includes the induction of classical interferon-stimulated genes as well as type III interferon and proinflammatory cytokines and chemokines like TNFα, IL-1α/β, IL-6, and CXCL-10 [185]. Interestingly, RIG-1 polymorphisms are linked to an increased risk of EV71-induced HFMD [186,187].

Toll-like receptor 3 (TLR3), located on the endosome membrane, is the third main sensor of viral double-stranded RNA. When bound to dsRNA, TLR3 associates with the Toll/interleukin-1 receptor (TIR)-containing adapter inducing IFN-β (TRIF or TICAM1). It activates IRF3 and NF-κB, triggering the activation of antiviral defense and inflammatory response in the infected cells. The TLR3/TICAM1 pathway is crucial for protecting the immune system against picornavirus infections. TICAM-1^−/−^ and human PVR—transgenic mice were more susceptible to poliovirus infection than the wild-type and MAVS^−/−^ transgenic mice [188]. Accordingly, TICAM-1^−/−^ transgenic mice exhibited the lowest levels of type I IFN mRNA in spleen dendritic cells among the mice with knockouts of various dsRNA sensors. Recent research indicates that the antiviral response to poliovirus infection includes pathways mediated by MDA5, TLR3, and the TLR adaptor MyD88, with TLR3 being the most profoundly upregulated [189].

RIG-I activity is regulated by the inhibition of Ubiquitin-Specific-Processing Protease (CYLD) expression, as well as by miR-526a. EV71 infection specifically enhances miR-526a expression in macrophages through an interferon-regulatory factor (IRF)-dependent mechanism. Virus-induced miR-526a activation and suppression of CYLD are both blocked by EV71 3Cpro, which is required for effective infection; ectopic miR-526a expression downregulates EV71 replication [190]. CVB3 activates another microRNA (miR-30a) to facilitate its replication. This is achieved through modulation of the interferon response, as miR-30a is a potent negative regulator of IFN type I signaling, acting on the tripartite motif protein 25 (TRIM25) [191]. Overexpression of TRIM25 markedly inhibits CVB3 replication, while TRIM25 knockdown leads to an increase in virus RNA production and titer and expression of the VP1 protein. By inhibiting TRIM25 expression and TRIM25-mediated ubiquitination of RIG-I, miR-30a reduces IFN-β production and activation. This may represent a mechanism by which CVB3 evades the host’s innate immune response [191,192,193].

## 11. MAVS

MAVS is a common adaptor molecule of RIG-I/MDA5, thus being essential for type I IFNs [194,195]. Its C-terminal transmembrane domain confers localization on the outer membrane of mitochondria [196]. Viral infection triggers the activation of RIG-I/MDA5, which, in turn, leads to the recruitment of MAVS through tandem Activation and Recruitment Domains (CARDs). Subsequently, MAVS connects RIG-I/MDA5 to IKK and TBK1/IKKε, activating IRF3 and NFκB—the two crucial components of the pathway that induce IFN in response to viral infection [197,198]. The interaction between RIG-I and MAVS activates several signaling pathways, including NF-κB and IRF [199,200]. These events initiate the production of type I interferons [201].

RNA viruses such as Sendai virus (SeV) and VSV trigger the activation of TRIM21, interacting with MAVS and catalyzing MAVS polyubiquitination, activating IRF3, and suppressing viral infections [202]. When mice are infected with CVB3, TRIM21 expression is increased [203]. Assessment of viral kinetics revealed that the cleavage of MAVS is a significant predictor of outcomes of infections. A common MAVS mutation that eliminates the protease-targeted region, ironically, increases rates of CVB3 infection [204]. Despite type I IFN signaling induced by CVB3 infection in HeLa cells, antiviral cytokines cannot restrict CVB3 replication. This can be rescued by the treatment of cells with exogenous MAVS protein before or after CVB3 exposure for enhancement of the innate immune response and prevention of virus spread and consequent cell damage [205].

Overexpression of MAVS in RD cells can induce both autophagy and apoptosis [206]. MAVS-induced apoptosis requires the inhibition of the extracellular signal-regulated kinase (ERK) signaling pathway and activation of c-Jun N-terminal kinase (JNK) signaling. Additionally, suppression of autophagy by 3-methyladenine (3-MA) enhances MAVS-induced apoptosis by increasing the cleavage of caspase-3 and poly(ADP-ribose) polymerase (PARP). Rapamycin-induced autophagy suppresses apoptosis by MAVS overexpression. Many viruses target MAVS for evasion from the innate immune response. One of the first described examples was the cleavage of MAVS by hepatitis C virus serine protease [207]. Later, such cleavage was shown for EV71, Echovirus 7, and CVB5, that target the Gly209, Gly251, and Gly265 residues of this cellular protein [208]. Other enterovirus strains cleave MAVS at different sites, as exemplified by CVB3 targeting the Gln148 residue [209]. Coxsackievirus A16 (CA16) may also suppress MAVS expression for efficient replication [206].

## 12. Sialic Acids

Sialic acids are nine-carbon carboxylated sugars. They are commonly found as moieties of glycoproteins and glycolipids at the surface of mammalian cells [210]. They play a significant role in the mediation of cell–cell contacts, the immune response, and host–pathogen interaction [211,212]. Concerning enteroviruses, the presence or absence of specific sialic acids on the surface of host cells can affect virus attachment, entry, and therefore virus tropism [5,213,214,215,216].

Many enteroviruses, including coxsackieviruses [217] and echoviruses, use sialic acid-containing molecules as cell surface receptors [218]. The ability of enteroviruses to bind sialic acids is primarily conferred by the pocket factor [177]. Some enterovirus strains have adapted to recognize variants of specific sialic acids for a more efficient infection of target host species/tissues [219]. The interaction between enteroviruses and sialic acids depends on evolutionary dynamics. Over time, the virus may undergo genetic changes to adapt to different sialic acid receptors, which might affect virus pathogenicity and tissue tropism [220,221]. This evolution may have significant implications for the emergence of new strains and the potential for interspecies transmission [222].

Understanding the role of sialic acids in enterovirus infection may have therapeutic implications. Targeting the interaction of viruses with sialic acids, such as blocking the binding site on the viral capsid, may be a potential strategy for developing antiviral drugs [5]. However, this field remains largely unexplored, warranting additional studies.

## 13. PLA2G16

PLA2G16, also known as the group XVI phospholipase A2 or AdPLA (lipid-specific phospholipase A2), is an enzyme belonging to the phospholipase A2 (PLA2) family [223]. PLA2 enzymes play a significant role in lipid metabolism [224], inflammation [58], and the regulation of various cellular processes. PLA2G16 has received attention in the context of enterovirus infection due to its potential role in the replication and pathogenesis of several enteroviruses [54,225].

PLA2G16 appears to be involved in virus reproduction by promoting the formation of membranes or vesicles that serve as platforms for viral RNA replication, possibly by modifying cellular lipids and their recruitment to replication organelles [225]. Moreover, this protein ensures the delivery of viral RNA into the cytoplasm [226]. In contrast, it cannot act as a viral receptor or a factor of virion trafficking into the cell. A genome-wide forward screen using Haplobank pointed to PLA2G16 as a factor essential for the cytotoxicity of rhinovirus A—a pathogen responsible for the common cold [227]. PLA2G16 knockdown reduced the migration and invasion of p53-null cells, while its overexpression enhanced their invasion. Mutant p53 elevated PLA2G16 levels in mouse and human osteosarcoma cells, suggesting PLA2G16 as a downstream target of p53 [228]. Beggen et al. reported the bioselection of an EV71 variant in PLA2G16-knockout cells; the mutations in the viral capsid protein reduced the dependency of the pathogen on PLA2G16, although at a cost of reduced thermostability [225].

In addition to its significance for enterovirus replication, PLA2G16 participates in unrelated cellular processes like lipid metabolism, adipocyte function, and stress responses. Understanding the specific mechanisms by which PLA2G16 promotes viral replication and pathogenesis in enterovirus infections remains an active research area. It is also important to note that PLA2G16′s involvement may vary across different enterovirus serotypes and strains [226].

## 14. Expression of the Receptors in Tumors

### 14.1. Poliovirus Receptor

Poliovirus receptor is a protein that is ubiquitously expressed in a variety of cells and tissues, albeit mostly at moderate levels [229,230]. However, many types of tumors exhibit elevated PVR expression compared to normal tissues. They include colorectal [231,232], prostate [233], renal [233], pancreatic [233,234], and esophagus small cell carcinomas. Higher levels of the receptor were also reported for non-small cell lung [235] and gastric [236] cancers, melanoma [237], liver cancer [238], and specifically cholangiocarcinoma [239], glioblastoma [240], bladder cancer [238,241] and its muscle-invasive type [242], and breast cancer [243] including its triple-negative variants [244]. PVR is likely to be overexpressed in neuroectodermal tumors, as it is controlled by the Sonic Hedgehog signaling pathway [245]. Indeed, several groups demonstrated elevated levels of PVR/CD155 in medulloblastoma [246,247]. Induction of PVR/CD155 is not a feature of advanced tumors only: increased expression could be already observed at a stage of precancerous lesions (at least in the stomach [236]) and early-stage tumors [248]. Importantly, the development of cancer in many cases is accompanied by the presence of soluble PVR/CD155 that can serve as a biomarker [244,249].

Increased expression contributes to the motility and invasiveness of tumor cells, leading to higher recurrence rates and, therefore, to poor prognosis, as described for different types of tumors [233,235,239,241,250,251,252,253]. In addition, high expression of PVR was noted as a negative prognosis factor for multiple myeloma [254]. High levels of the receptor are also associated with enhanced angiogenesis [252]. For triple-negative breast cancer, high PVR expression is a feature of tumor cells with the mesenchymal phenotype [255]. However, at least for medulloblastoma, no differences in PVR expression between primary tumors and metastases were shown [247]. PVR/CD155 also contributes to cancer progression by downregulating the immune response to tumor cells. For example, its presence on the surface of tumor cells leads to the inactivation of melanoma-specific T-cells [237] and suppression of NK-cells [256] that could have eliminated cancer cells. In case of melanoma, PVR expression was shown to confer resistance to anti-PD1 immunotherapy [257]. High expression of PVR can also trigger the degradation of CD226 on the surface of CD8+ T-cells, also leading to an immunosuppressed phenotype [258].

### 14.2. ICAM-1

ICAM-1 is expressed in various types of tumors. Most reports discuss high levels of ICAM-1 in lung cancer, specifically in its triple-negative variant [259,260,261] as well as its metastasis into lungs [262]. Its expression is also elevated in thyroid carcinomas [263,264,265], although not in all tumors [266]. Eighty-three per cent of clear cell renal carcinomas [267] and half of gastric tumors [268] are ICAM-1-positive, although metastasis of the latter into liver leads to enhanced expression in almost all cases. A similar pattern was reported for colon [269] and bladder [270] cancer: expression of ICAM-1 is associated with invasiveness and poor prognosis. The opposite dependence between ICAM-1 expression and invasiveness was described for melanoma, in which the presence of this receptor on tumor cells is associated with lower aggressiveness and a higher patient survival rate [271,272]. Indeed, metastatic derivatives of melanoma cells are negative for ICAM-1 [271].

ICAM-1 is also found in tongue squamous cell carcinoma, at least in its invasive front area [273]. Not much is known about the status of ICAM-1 in lung tumors, but most, if not all, non-small cell lung cancer cell lines express this receptor [274]. In contrast, cell lines corresponding to small-cell lung cancer are predominantly ICAM-1-negative. Bladder cancer is characterized by varied levels of ICAM-1 and, as a consequence, different sensitivities of tumors to Coxsackievirus type A [275]. In contrast, ICAM-1 is not expressed in retinoblastoma [276]. It is also reduced in ovarian adenocarcinoma, which is achieved via methylation of its promoter [277].

There are also several reports showing an increase in ICAM-1 expression in cancer cells after treatment with pharmacological agents or experimental approaches, thus providing a clue to the enhancement of oncolysis. In B-cell lymphoma cells, the increase could be triggered by metformin [278], while in osteosarcomas, by interleukin 6 [279].

### 14.3. SCARB2

SCARB2 was cloned in 1991 and described as LIMP2 [280]. This protein is localized on the ER and plasma membrane, at least in Cos cells [280], as well as on the membranes of lysosomes in various types of cells [281]. SCARB2 mediates cholesterol efflux [282] and, together with SR-BI, participates in the influx of high-density lipoproteins [283]. Again, both SR-BI and SR-BII mediate HCV entry [284].

SCARB2 is highly expressed in the spleen, at somewhat lower levels in liver, colon, and adrenal gland, and at low levels in the heart, lung, and thymus [283]. No expression was found in the kidneys and pancreas [283]. Evaluation of its levels in tumor cell lines revealed high expression in cervix carcinoma HeLa cells, melanoma 14Mel and C32 lines, and promonocytic leukemia U973 cells. Moderate expression was noted in promyelocytic (HL60) and erythro-(HEL) leukemia cell lines, whereas very low levels of expression were described for a Jurkat cell line derived from a patient with T-cell leukemia [281]. In patient samples, elevated expression compared to normal tissues was reported for glioblastoma [285], breast cancer [286], and lymph node-positive oral squamous carcinoma [287], with high levels correlating with poor prognosis. Unfortunately, information about its expression in other types of cancer is still missing.

### 14.4. CAR

CAR is expressed in a wide array of normal tissues, while in tumors its levels remain heterogenous [288]. It is highly expressed in ovarian cancer [289,290,291], squamous cell and small cell lung cancer [292,293,294,295], oral squamous cell carcinoma [296], thyroid [297], prostate [298,299], and hormone-sensitive breast cancer [300,301,302], with levels often higher that in the respective normal tissues. CAR is also often found in soft tissue and bone sarcomas [296,303,304,305], although contrary data are also present [306]. Specifically, in prostate tumors CAR is found on the cell membrane, indicating that it should be fully functional [298]. This implies that these types of cancer could be treated with viruses that are internalized by this receptor. However, in the case of ovarian carcinoma, the highest levels are observed in early-stage tumors, while late-stage disease is characterized by a decline in CAR levels [307]. Nevertheless, the recurrent tumors are still receptor-positive, indicating that oncolytic viruses could still be effective [290], pending that an increase in soluble CAR receptors from an advanced tumor will not diminish the levels of the virus [291]. Endometrial sarcomas, another type of gynecological cancer, are in 50% of cases CAR-negative, although some positive tumors exhibit high levels of expression of this receptor [308]. Heterogenous expression was also described for head and neck cancer [309,310]. In non-small cell lung cancer, the expression of CAR is a feature of cancer stem cells [294] that are tumor-initiating cells and the cells with the tumor-resistant phenotype. This also points to the respective oncolytic viruses as a possible treatment for the disease. However, as tumors also produce soluble CAR, this can lead to reduced efficiency of oncolysis due to virus neutralization [311].

In contrast, pronouncedly decreased CAR expression was reported for bladder [312,313] and prostate [288] cancer, as well as in various gastrointestinal tumors, including esophagus [314,315], gastric [316], pancreatic [314], liver [314,317], and colon [288,314,318,319] cancers. However, CAR expression is elevated in Barrett’s esophagus, though in advanced tumors the receptor is no longer detected [320]. In bladder cancer and astrocytomas, CAR levels continue to decline with tumor progression [312,321], and in many bladder tumors and cell lines CAR is no longer detected [313,322,323]. Most advanced astrocytomas (glioblastomas) [321,324,325] and invasive gastric tumors [316] do not express the CAR receptor. No CAR expression was detected in B16 melanoma cell lines [326].

As decreased expression of the CAR receptor results from the methylation of its promoter, the inhibitors of histone deacetylases restore/enhance its levels [301,327]. However, hypoxia is another factor that reduces CAR levels, at least in prostate, gastric. and colon cancer [328]. In estrogen receptor-positive breast cancer cells, CAR expression could also be increased by treatment with estradiol [302], while in glioblastomas and cervical cancer CAR levels are reduced during treatment with dexamethasone [329].

### 14.5. DAF

DAF has heterogenous expression in various types of cancer. For example, it is not expressed in ductal carcinoma [330], meningiomas and astrocytomas [331,332], neuroblastomas [333], and gliomas [334], and many cases of non-Hodgkin lymphoma [335] but is abundant in acute lymphoblastic (ALL) [336] and chronic myeloblastic (CML) [336] leukemias and in many cell lines derived from Burkitt’s lymphoma [337]. Another group of researchers nevertheless noted that its expression in ALL and CML is decreased compared to cells from healthy donors [338]. Increased expression of DAF has been reported for oral carcinoma [339] and Barrett’s esophagus that precedes esophagus carcinoma [340,341]. Tumors of the gastrointestinal tract are generally positive for DAF, as noted for colon [342], pancreatic [342,343], and gastric [342,344] cancers, with the exception of KATO III gastric cancer [342]. This does not imply that DAF is present in all tumors of the abovementioned types of cancer: for colon cancer, approximately 30% of tumors remain negative [132], including metastasis of liver cancer [345]. But, there is a clear increase in DAF expression in tumors compared to normal tissues and benign tumors [132,346]. A similar increase was also reported for gastric [347] and pancreatic [343] cancer, as well as osteosarcoma [347], cholangiocarcinoma [348], gallbladder cancer [349], and oral squamous cell carcinoma [350]. In the case of gastric cancer, this increase is attributed to *Helicobacter pylori* and specifically to its CagA antigen [351,352].

Thyroid cancer is another type of malignancy that is associated with elevated DAF expression [353,354]. DAF expression was also reported for breast [355] and renal [356] cancer, head and neck squamous cell [357,358] and nasopharyngeal [359] carcinomas, as well as in various gynecological cancers: cervix squamous cell carcinomas [360], endometrial cancer [361,362,363], and uterine serous carcinomas [364]. The only exception is ovarian cancer, for which DAF expression is decreased [361]. A noteworthy point is that the increase in DAF expression in endometrial cancer is observed in early-stage tumors, whereas at advanced stages the expression of the receptor diminishes [362]. Finally, DAF is expressed in lung cancer cell lines [365].

Importantly, high DAF expression has been associated with invasiveness and poor prognosis, as exemplified for colon [343,366], cervix [360], gallbladder [99], and nasopharyngeal [359] cancer.

It should be noted that in some types of cancers, DAF is not expressed in all cells within a tumor. In neuroblastomas, DAF is found only in a minor population of tumor cells, with significant co-staining with HIF2α pointing to hypoxia as one of the mechanisms of induction of this receptor [367]. Similar heterogeneity in expression within a tumor was reported for endometrial cancer [368]. In gliomas, the weak staining was noted not in tumor cells but in endothelia [334], while in cervix carcinomas the DAF is found in stromal cells adjacent to tumor cells [369].

### 14.6. KRM1

There are only scarce data about KRM1 expression in various types of tumors and cancer cell lines. The Protein Atlas suggests that this gene has moderate-to-low expression in almost all cell lines investigated, with the highest rates of its transcript registered in the Rhabdomyosarcoma Ch30 cell line, gastric cardia OE19 cells, hepatocarcinoma HepG2, and Sk-Mel-30 melanoma cells [229,230]. The same database confirms the presence of the protein in Ch30 cells, as well as in the H2OS osteosarcoma cell line, albeit with nuclear localization. This gene is efficiently transcribed in the lung adenocarcinoma A549 cell line, although other lung cancer cells may exhibit much lower expression levels [370]. Similarly, KRM1 expression was shown for prostate and breast cancer cell lines (such as PC3, MCF7, or T47D) whereas such cell lines as PC3 and MDA-MB-231 are characterized by significantly lower levels of this gene [371]. Sumia et al. reported the reduced expression of the KRM1 gene in breast invasive carcinoma, colon carcinoma, head and neck squamous cell carcinoma, kidney renal carcinomas, prostate adenocarcinoma, and thyroid carcinoma, compared to normal tissues [372]. The same team showed elevated expression in hepatocellular carcinoma and cholangiocarcinoma, as well as in lung squamous cell carcinoma. Elevated expression was also reported in stromal cells from patients with multiple myeloma, compared to stromal cells from healthy patients [373].

### 14.7. FcRn

FcRn is expressed at high levels in various organs and tissues. The Protein Atlas reports that the highest levels are in the liver, spleen, and gastrointestinal tract, while the lowest are in the retina and cerebellum [229,230]. However, its expression is significantly diminished in various types of cancer. Lung, bladder, ovarian, and cervix cancer, as well as head and neck squamous cell carcinomas are FcRn-negative [374]. This gene is not expressed in most breast tumors, including triple-negative breast cancer, and also not in pancreatic and renal tumors. FcRn expression is detectable in 50% of cases of colorectal cancer [374] or in 40% of endometrial cancer. Non-small cell lung cancer is also mostly negative for the expression of this gene, although FcRn-positive cases are considered to have better survival rates [375]. Moreover, the detection in such tumors is often found not in cancer cells but in resident and tumor-infiltrating immune cells [163]. In addition, FcRn is expressed in dendritic cells that are involved in the protection against colorectal cancer [153]. FcRn expression is elevated in pilocytic astrocytomas—the benign tumors with low growth rates [376]. FcRn expression was reported in hepatocellular carcinoma cell lines (HepG2 and Snu-475) [377], as well as in THP1, Jurkat, and U937 cells that were derived from patients with acute monocytic leukemia, T-cell leukemia, and histiocytic lymphoma, respectively [378]. In contrast, various breast and prostate cancer cell lines are FcRn-negative [164].

## 15. Conclusions

The intricate interplay between enteroviruses and host cell factors offers a unique window into the mechanisms of viral infection and its potential exploitation for therapeutic purposes, particularly in cancer therapy. The specificity of interaction of enteroviruses with cellular receptors such as PVR, DAF, ICAM-1, and SCARB2 not only determines the tropism and pathogenicity of these viruses, but also unveils a promising avenue for oncolytic virus therapy. The ability of certain enterovirus strains to selectively target and lyse tumor cells emphasizes their therapeutic potential as anticancer agents. This could be exemplified by the use of Rigvir in clinical practice for treatment of melanoma as well as by ongoing trials of other enteroviruses.

Evaluation of the expression of these receptors in tumors gives clues to the choice of oncolytic virus for its treatment. SCARB2-expressing hepatocellular carcinoma cells or ICAM-1-positive triple-negative breast cancer cells could be targeted by EV71 or Coxsackievirus A, while PVR-expressing glioblastomas could be targeted by polioviruses. Special emphasis should be given to DAF, as this receptor is required for fast and lytic echovirus or coxsackievirus (A or B) infections. In the latter case, oncolytic therapy should probably not be applied to patients on non-steroid anti-inflammatory drugs, as they can diminish expression of the DAF receptor. Moreover, the influence of antitumor agents on the expression of receptors for enteroviruses merit further studies. We should also mention that tumors could possibly be treated by a composition of enteroviruses, as tumors are heterogeneous and could be composed of cells with different patterns of receptor expression.

Another question that requires future study is the exploration of whether enteroviruses can establish non-lytic infection, as there are several reports of their non-lytic replication [2,379]. Similar reports of high-level infection in tumor cells in the absence of signs of cytotoxicity also exist for such viruses as SARS-CoV-2 [380,381]. Such infection would not only be inefficient but may also impose threat to cancer patients. An investigation into mechanisms by which the virus may evade oncolytic [action and establish continuous replication is definitely required.

As we continue to unravel the complexities of enterovirus biology and its interaction with host cell factors, it is imperative that we also focus on the translational aspects of this research. Collaborative efforts at the interface of virology, molecular biology, immunology, and clinical oncology are essential to ensure host-cell usage of full therapeutic potential of enteroviruses against cancer. As a part of a broader oncolytic virus arsenal, enterovirus-based therapies offer hope for cancer patients, opening a new era of targeted, effective, and innovative treatments.
